# Enhanced detection and study of murine norovirus-1 using a more efficient microglial cell line

**DOI:** 10.1186/1743-422X-6-196

**Published:** 2009-11-10

**Authors:** Courtney Cox, Shengbo Cao, Yuanan Lu

**Affiliations:** 1 Department of Microbiology, College of Natural Sciences, University of Hawaii at Manoa, Honolulu, HI 96822, USA; 2 Department of Public Health Sciences, John A. Burns School of Medicine, University of Hawaii at Manoa, Honolulu, HI 96822, USA

## Abstract

**Background:**

Human Noroviruses are the predominant cause of non-bacterial gastroenteritis worldwide. To facilitate prevention and control, a norovirus isolated from mice can provide a model to understand human noroviruses. To establish optimal viral infectivity conditions for murine noroviruses, several cell lines of hematopoietic lineage, including murine BV-2, RAW 264.7, and TIB, as well as human CHME-5, were tested comparatively for their sensitivity to murine norovirus-1.

**Results:**

Except for CHME-5, all three murine-derived cell lines were susceptible to MNV infection. Viral infection of these cells was confirmed by RT-PCR. Using both viral plaque and replication assays, BV-2 and RAW 264.7 cells were determined to have comparable sensitivities to MNV-1 infection. Comparisons of cell growth characteristics, general laboratory handling and potential in-field applications suggest the use of BV-2 to be more advantageous.

**Conclusion:**

Results obtained from these studies demonstrate that an immortalized microglial cell line can support MNV-1 replication and provides a more efficient method to detect and study murine noroviruses, facilitating future investigations using MNV-1 as a model to study, detect, and control Human Norovirus.

## Background

Noroviruses belong to the family *Caliciviridae *and are a group of small, icosahedral, non-enveloped, positive-strand RNA viruses [1-6]. Most norovirus genomes range from 7.7-7.9 KB and contain three highly conserved open reading frames (ORF)[3]. Human Norovirus (HNV) strains are the predominant cause of non-bacterial gastroenteritis worldwide and are primarily transmitted through the fecal-oral route, usually by the consumption of contaminated food or water [1-3,7-12].

Despite worldwide occurrence and high level of incidence, there are no drug treatments or vaccines available to date. In fact, little is known about human norovirus biology due to the lack of a cell culture system or small-animal model for use in studies [7,9,13-16]. To facilitate the prevention and control of this human pathogen, a norovirus isolated from murine animals is currently considered as a model to understand human norovirus replication, life cycle, pathogenesis, and host immune response [7,14]. Recent studies have demonstrated that MNV-1 and human norovirus share many biochemical and genetic characteristics, including their genome, genomic organization and function, virion size (28 to 35 nm in diameter), shape, and buoyant density [16], induced symptoms [16], and transmission in nature, primarily via the fecal-oral route [16]. In particular, murine norovirus is known to be the only isolate among the five noroviral genogroups to replicate in cell culture and in small animals (mice), making it an excellent candidate as an experimental model for human norovirus [7,14,16].

The MNV-1 model has already provided some insights into norovirus biology. It was discovered that noroviruses possess a tropism for macrophages and dendritic cells during replication [7,16]. Wobus et al. (2004) showed that MNV-1 replicates readily in cell lines with a hematopoietic lineage, including the RAW 264.7 cell line, as well as in primary bone-marrow derived macrophages and dendritic cells. While it has been shown that other macrophage and dendritic cell lines, including IC21, P388D1, WBC264-9C and JAWSII, can also support MNV-1 replication [16], RAW 264.7 cells currently represent the most widely utilized immortalized cell line for MNV studies.

To facilitate the development of MNV-1 as a model for human norovirus, it is important to establish optimized *in vitro *laboratory conditions for MNV-1 infection and detection, including testing and identifying other hematopoietic cell lines for their susceptibility to MNV-1. This study describes a comparative test and evaluation of four readily available cell lines of hematopoietic lineage, including murine-derived microglial BV-2, murine-derived macrophages RAW 264.7 and TIB, as well as human-derived microglial CHME-5, for their potential use in detecting and studying MNV-1, and identifies a murine microglial cell line (BV-2) as a novel cell culture system for studying MNV-1, thus extending the current use of MNV-1 as a model for human norovirus.

## Methods

### Virus

Murine norovirus (MNV-1, generously provided by Dr. Philip C. Loh, The University of Hawaii Manoa) was initially propagated in RAW 264.7 cells. Cells were seeded into a TC-75 cm^2 ^flask so that an approximately 80-90% cell monolayer formed within 24 hours. Immediately prior to infection, all medium was removed and 250 μL of previously made viral stock in 2 mL of serum-free medium was added into the flask. The flask was incubated for 1 hour at 37°C with 5.0% CO_2_, and then washed twice with serum-free medium. Following the two washes, 10 mL of medium supplemented with 5% fetal bovine serum (FBS) (HyClone, Logan, UT) was added into the flask. The flask was then incubated for 48 hours, until approximately 90% viral-induced cytopathic effects (CPE) (rounding of cells, loss of contact inhibition and cell death) were observed. The flask was then stored at -80°C. After a 24-hour storage, the flask was then allowed to thaw at room temperature (RT). Following an additional freeze-thaw cycle, the content of the flask was completely removed and centrifuged at 3000 rpm for 5 minutes to remove all cellular debris. Supernatant was then removed and aliquoted into 1.5 mL microfuge tubes at 0.5 mL/tube. The viral aliquots were stored long-term at -80°C.

### Cell cultures

Four cell lines with a hematopoietic lineage, including murine BV-2 (Provided by Dr. Paul Jolicoeur, Université de Montréal), RAW 264.7 (ATCC Manassas, VA) and TIB (ATCC Manassas, VA), as well as human CHME-5 (Provided by Dr. Pierre Talbot, Université du Québec), were used throughout these experiments. All cell cultures were grown in high-glucose Dulbecco's modified eagle's medium (DMEM) (Sigma-Aldrich St. Louis, MO), supplemented with 10% FBS, penicillin (100 IU/mL) and streptomycin (100 μg/mL) (Sigma-Aldrich St. Louis, MO). Defined FBS (HyClone, Logan, UT) was used for the RAW 264.7 and TIB cell lines, while standard FBS (HyClone) was used for the BV-2 and CHME-5 cell lines. Cells were grown and maintained according to standard animal cell culture protocols and kept at 37°C with 5% CO_2_.

### Viral infection

Twenty-four hours prior to infection, cells in the exponential growth phase were harvested and seeded into 75 cm^2 ^tissue culture (TC-75) flasks at a density that allowed for a single cell monolayer within 24 hours. Immediately before infection, medium was removed and cells were washed twice with DMEM containing no FBS (DMEM-0). MNV-1 in serum-free high-glucose DMEM was added to each flask giving a multiplicity of infection (MOI) of 2. Cells were incubated for one hour at 37°C with 5% CO_2_. Flasks were rocked gently every 15 minutes for equal viral distribution. At the end of the one-hour adsorption, the inoculum was removed and replaced with 10 mL of the high-glucose DMEM containing 5% FBS (DMEM-5). The cells were then incubated for three days at 37°C with 5% CO_2_. Photomicrographs were taken on a daily basis to document specific viral-induced cytopathic effects (CPE).

### RNA Isolation and RT-PCR

Forty-eight hours after infection, cells were collected, washed twice with Dulbecco's phosphate buffered saline (DPBS) (Sigma-Aldrich St. Louis, MO), and pelleted by centrifugation. RNA isolation was performed with the RNeasy Mini Kit (Qiagen, Valencia, CA), according to the manufacturer's instructions. Isolated total RNA was prepared in a final concentration 5 μg/ml.

In order to confirm susceptibility of test cell cultures to MNV-1 infection, reverse-transcriptase polymerase chain reaction (RT-PCR) was performed using newly-designed oligonucleotide primers specific to the MNV-1 genome (Forward 5'-ATCGTGCTGAGCTGTGATTG {M1} and Reverse 5'-GTCAAGAGCAAGGTCGAAGG {M2}, NC-008311 Genbank). cDNA was initially generated using SuperScript II Reverse Transcriptase (Invitrogen, Carlsbad, CA) and random hexamers (Invitrogen, Carlsbad, CA), according to the manufacturer's instructions. PCR was then performed using the primer pair with an initial denaturing at 94°C for 5 min., followed by 35 cycles of denaturing at 94°C for 1 min., annealing at 60°C for 1 min., and extension at 72°C for 1 min., and then a final extension at 72°C for five minutes. Upon completion of the PCR reaction, samples were held at 4°C and then subjected to 2% agarose gel electrophoresis, along side a 100 bp marker (Perfect DNA™ 100 bp Ladder, Novagen, Gibbstown, NJ). Cell lines determined to be susceptible to MNV-1 were used for further testing.

### Plaque assay

The sensitivity of three murine microglial cells to MNV-1 was quantitatively compared using a newly established viral plaque assay protocol developed in this laboratory. In brief, cells were seeded into 6-well plates at a rate of ~7.5 × 10^4 ^cells/well for the BV-2 cell line and ~5.5 × 10^5 ^cells/well for the RAW 264.7 cell line, densities that allowed the formation of a confluent monolayer within 24 hours. Immediately before MNV-1 infection, a series of 10-fold dilutions of a MNV-1 stock prepared in DMEM-0 were similarly inoculated onto the three murine cell lines grown in the 6-well plates, following aspiration of the medium and two cell washes with DMEM-0. Plates were then incubated for one hour at 37°C in a humidified 5% CO_2 _incubator, with gentle rocking every 15 minutes to allow even distribution of the viral inoculum. All liquid was removed from the plates and cells were covered with 2 mL/well of a 1.5% methylcellulose overlay medium. After three days, cells were fixed and stained with 2 mL/well of crystal violet-formalin solution for eight hours. Plates were vigorously washed with tap water and viral induced plaques were counted. Titers were calculated and compared between the different cell cultures.

### Replication Assay

In addition, MNV-1 replication in the BV-2 and RAW 264.7 cell lines was also comparatively analyzed using a single-step viral replication assay. Briefly, BV-2 and RAW 264.7 cells at their exponential phase were harvested as previously described, and seeded into 24 separate T-25 cm^2 ^tissue culture flasks (Cellstar Greiner Bio-One, Monroe, NC), 12 per group, at a rate of 1.8 × 10^5 ^cells/flask in 5 mL DMEM cell growth medium. Cells were then incubated at 37°C with 5% CO_2 _to allow cell monolayer formation. After a 24-hour incubation, medium was removed from the flasks and cells were washed twice with serum-free medium DMEM-0. Diluted MNV-1 in serum-free high-glucose DMEM was added to each of the flasks, giving a final MOI of 2.0. Cells were then incubated at 37°C with 5% CO_2 _for 48 hours. Every four hours, one flask per infected cell group was removed from the incubator. The medium from each flask (representing the extracellular virus) was collected and stored separately in a 15 mL centrifuge tube at -80°C. Cells attached to each flask (representing the intracellular virus) were washed twice with sterile DPBS, and also stored at -80°C. This process was repeated until a total of 12 samples over a 48-hour time period were taken for each infected cell line. Viral titers of both extracellular and intracellular virus at each time point were determined by plaque assay (as previously described) and totaled.

## Results

### Susceptibility of cells to infection by MNV-1

**Figure 1:**
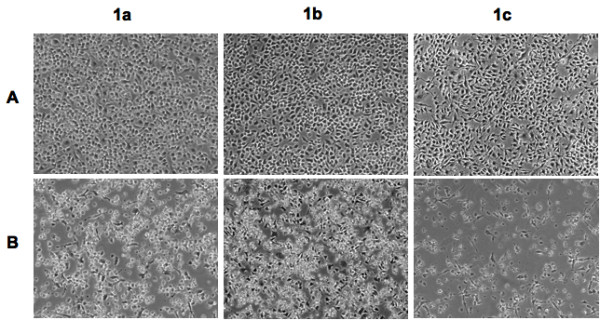
**Susceptibility of cells to MNV-1 infection**. Four cell lines BV-2 (**1a**), RAW 264.7 (**1b**) and TIB (**1c**) were infected with MNV-1. Photomicrographs were taken to visualize CPE at selected post-infection times: cells pre-infection (A) and extensive CPE at 72 hours post-infection (B). Original magnification was at 100×.

Four cells lines of hematopoietic lineage were tested comparatively for their sensitivity to MNV-1 infection. Their viral susceptibility was evaluated by both the appearance of viral-induced CPE (rounding of cells, loss of contact inhibition, and cell death) and the production of infectious viral particles. The results show that all three murine-derived cell lines are susceptible to MNV-1 infection. Viral-induced CPE was observed in the BV-2 cell line as early as 16 hours post-infection, with 100% CPE occurring after three days (Figure 1). Infection of the RAW and TIB cells showed signs of visible CPE at 18 hours post-infection, and also had 100% CPE occurring 3 days post-infection (Figure 1). However, the human-derived CHME-5 cell line appeared refractory to MNV-1 infection, as no viral-induced CPE was observed at any time period (data not shown).

**Figure 2:**
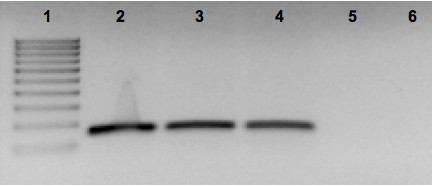
**RT-PCR**. Confirmatory detection of MNV-1-infection of test cells. Total cellular RNA isolated from MNV-1 infected cells was subjected to the RT-PCR amplification as described in method section. Results show that the BV-2, RAW 264.7 and TIB cell lines were infected by MNV-1 (as evident by the appearance RT-PCR product), while CHME-5 appears to be refractory to MNV-1 infection. Lanes: 1 = 100 bp marker, 2 = MNV-infected BV-2 cells, 3 = MNV-infected RAW 264.7 cells, 4 = MNV-infected TIB cells, 5 = infected CHME-5 cells, and 6 = Negative control (ddH_2_O).

In order to confirm susceptibility to MNV-1 infection, RT-PCR was performed on RNA isolated from all four MNV-1 infected cell lines, using primers designed to amplify a conserved portion of the MNV-1 genome. Results confirmed that all three murine-derived cell lines are susceptible to MNV-1 infection (as evident from the appearance of the expected RT-PCR products) and that the human-derived CHME-5 microglial cell line is refractory to MNV infection (Figure 2). Representative amplicons were sequenced and shown to have a 100% sequence identity to the respective genomic portion of MNV-1 (data not shown). To ensure accurate results, the experiment was performed in triplicate.

### Sensitivity of cells to infection by MNV-1

**Figure 3:**
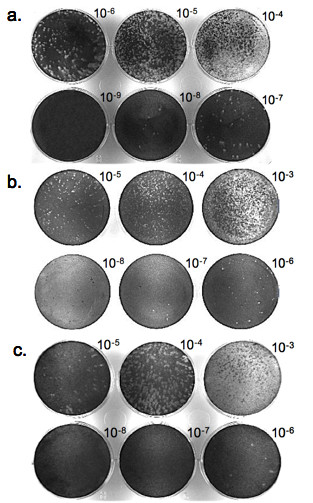
**Comparative plaque assays for MNV-1 detection**. Cells were infected with same preparation of diluted MNV-1, maintaining a consistent MOI. Following a one-hour adsorption period, infected cultures were covered with a 1.5% methylcellulose overlay medium. Cells were then stained with a crystal violet-formalin solution at day 3 post infection and titers were calculated. **A**. BV-2 plaque assay; **B**. RAW 264.7 plaque assay; and **C**. TIB plaque assay.

**Figure 4:**
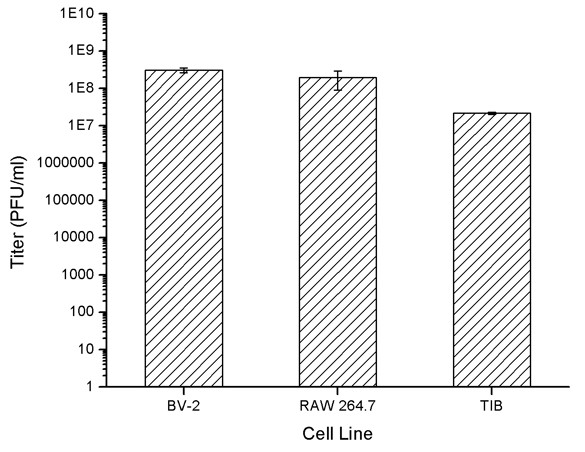
**Comparative graph of plaque assay results**. BV-2 produced a titer of 3.1 × 10^8 ^pfu/mL. The RAW 264.7 cell line produced a comparable viral titer of 1.93 × 10^8 ^pfu/mL. The TIB cell line produced a titer of 2.17 × 10^7 ^pfu/mL;

In order to compare the sensitivities of the susceptible cell lines to MNV-1, both viral plaque and replication assays were performed. Plaque assay data (Figure 3a) shows that when infected with the same viral stock, BV-2 appears to be the most sensitive cell line to MNV-1 detection, with a titer of 3.6 × 10^8 ^pfu/mL (Figure 3b), followed by RAW 264.7 cell line, with a comparable viral titer of 1.5 × 10^8 ^pfu/mL (Figure 3c). The TIB cell line appeared to be the least sensitive cell line to MNV-1 infection, producing a titer of 2.2 × 10^7 ^pfu/mL (Figure 4), one order of magnitude lower than the other two cell lines.

**Figure 5:**
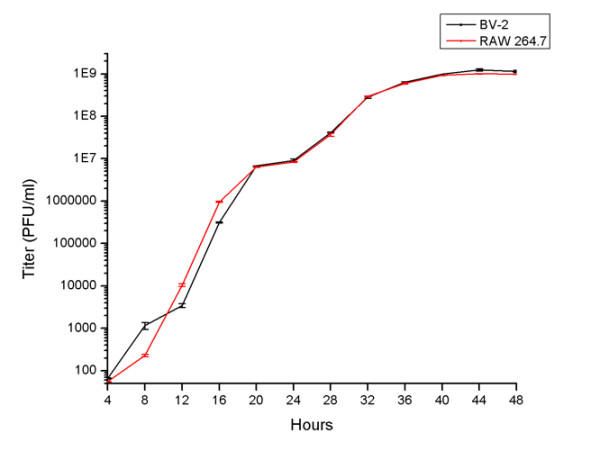
**Replication Assay**. Cells were infected with a MOI of 2 and allowed to incubate for 48 hours at 37°C with 5% CO_2_. Samples were taken every four hours and viral titers were determined by plaque assay. The replication curve of MNV-1 in both the BV-2 and RAW 264.7 cell lines was then plotted. Results are showing similar growth and viral titer production in both cell lines, among all time points.

To further confirm these findings, a single-step replication assay for MNV-1 grown in both the BV-2 and RAW 264.7 cell lines was performed. These two cell cultures were infected with a same MNV-1 stock and incubated for 48 hours, with samples of both intracellular and extracellular virus being taken every 4 hours. MNV-1 titers were calculated for each time point using plaque assays, and a growth curve of total MNV-1 replication in each cell line was plotted. As seen in Figure 5, both curves show a short eclipse period of viral replication, with MNV-1 replication becoming detectable at 4 hours post-infection. In both cell lines, replication continued exponentially until 40 hours post-infection, at which time it reached a constant viral titer of over 10^9 ^pfu/mL (Figure 5). Comparison between the two viral replication curves shows that MNV-1 begins to replicate within the same time period in both cell lines and reaches similar end-point titer values. In sum, these data confirm that the BV-2 and RAW 264.7 cell lines are generally equal in their susceptibility to MNV-1 infection, and their ability to produce virus particles.

## Discussion

Currently, there is no cell culture or small animal model available for studying human norovirus strains, thus presenting a need to find an appropriate model [7,9,13-16]. MNV-1 is an excellent candidate model to study norovirus biology because of its similarities to human noroviruses [16], as well as the fact that it replicates in both cell culture and small animals [7]. Establishment and optimization of this model are important, as it provides an opportunity to study norovirus biology in a natural host. Previous studies of MNV-1 have shown that murine norovirus has a tropism for macrophage and dendritic cells, leading to the development of the first cell culture and plaque assay systems for norovirus [7,14]. In order to further establish the use of MNV-1 as a model, these experiments were performed to test and compare four readily available cell lines for their susceptibility and sensitivity to MNV-1 infection.

Initially, four cell lines were tested for their susceptibility to MNV-1 infection by direct infection of these cells with MNV-1 stock. The results showed that all three murine-derived cell lines were susceptible to MNV-1 infection, as evident by the appearance of viral induced CPE in the infected cell cultures, which became more extensive with time. MNV-1-infection of these cell cultures was then confirmed by the positive detection of the target MNV-1 gene from the infected cells using RT-PCR and subsequent sequence analysis of the representative amplicons, which showed a 100% sequence identity to the MNV-1 genome.

The sensitivities of the three susceptible cell lines to MNV-1 were then compared using a newly modified viral plaque assay protocol established for optimal quantitation of infectious MNV-1. Plaque assays using an agarose or agar overlay medium have been used reported for MNV-1 infection studies, followed by staining with a neutral red dye [7]. However, these methods can be inefficient, especially when plaque assays are performed in large quantities, as both agarose and agar harden quickly, thus creating the need for multiple heat/thaw methods. Therefore, a plaque assay system with a methylcellulose-based overlay medium was tested and developed. A 0.75% methylcellulose overlay medium, which is commonly used for marine viral titration, was initially used. However, the MNV-1-induced plaques formed under these conditions appeared very smeared and indistinct, suggesting an ineffective localization of MNV-1 replication at 37°C. This observation led to the serial increase of methylcellulose concentration to 1.5%, which was found to be sufficient to contain viral infection to foci, allowing for distinct plaque formation.

The comparative plaque assay results showed that BV-2 cells were at least equally sensitive to MNV-1 detection as compared to RAW 264.7 cells, the currently used model for the virus. To further compare the sensitivity of BV-2 with RAW 264.7 cells, a single-step MNV-1 replication assay was performed in both the BV-2 and Raw 264.7 cells. The results obtained from this test showed that the MNV-1 replication curve in RAW 264.7 cells is consistent with those observed in previous replication studies [17]. This data also showed that the replication curves between the RAW 264.7 and BV-2 cell lines looked very similar in nature, with endpoint viral titers appearing equivocal between these two cell lines. These findings further confirm previous results that, like RAW 264.7 cells, the BV-2 cell line is very sensitive to MNV-1 infection and represents a new *in vitro *system for studying MNV-1.

As compared to RAW 264.7 cells, the BV-2 cell line has shown to be a more efficient cell culture system to be used for MNV-1 studies. BV-2 cells are known to grow quickly and can be resilient. This allows for quick cell propagation and eliminates the need for more challenging cell passage methods such as cell scraping, as needed for RAW 264.7 cells. Instead, basic cell culturing methods like trypsinization can be used regularly. In addition, the BV-2 cell line is very easy to maintain and has fewer nutritional requirements and does not secrete cellular toxins that interfere with cellular growth and medium pH levels (unpublished observation), whereas constant care is required for the RAW 264.7 cells. Lastly, the larger cell size of BV-2 makes it more useful than RAW 264.7 cells as a simple detection system. The presence of virus in unknown samples, such as environmental samples, could be more easily detected in the BV-2 cell line than in an identical assay using the RAW 264.7 cell line. The larger cell size allows for the use of less BV-2 cells than RAW 264.7 cells in identical assays, yielding a higher MOI. As MOI is important for viral infectivity, having a higher MOI in BV-2 than in RAW 264.7 cells is highly beneficial. This strongly suggests that samples containing smaller amounts of virus could still be tested in the BV-2 assay, while possibly falling below detectable levels in an identical assay utilizing RAW 264.7 cells. Overall, these findings support the use of BV-2 as a more efficient cell culture system to study MNV-1.

## Conclusion

These data show that two new murine-derived microglial/macrophage cell lines are sensitive to MNV-1 infection and support the use of the BV-2 cell line in future studies of MNV-1. While future research should also involve testing other murine microglial and macrophage cell lines for increased sensitivities to MNV-1, present research has established new laboratory techniques that allow the use of an alternative cell line to study MNV-1. Inaddition, testing this cell line for its susceptibility to other MNV strains will be beneficial in determining the utility of this cell line as a model to study norovirus. This cell line, as well as undiscovered cell lines, will lay the essential groundwork for future investigations to establish the use MNV-1 as a model to study, detect, and control human noroviruses.

## Competing interests

The authors declare that they have no competing interests.

## Authors' contributions

CC carried out all experimental testing and drafted the manuscript. SC participated in the design of the study by providing technical assistance to CC regarding plaque assays. YL conceived of the study, and participated in its design, coordination, and data analysis and manuscript revision. All authors read and approved the final manuscript.
